# Novel optimization technique of isolated microgrid with hydrogen energy storage

**DOI:** 10.1371/journal.pone.0193224

**Published:** 2018-02-21

**Authors:** Eman Hassan Beshr, Hazem Abdelghany, Mahmoud Eteiba

**Affiliations:** 1 Department of Electrical and Control Engineering, Arab Academy for Science, Technology and Maritime Transport, Cairo, Egypt; 2 Department of Electrical Power and Machines, Fayoum University, Fayoum, Egypt; Universita degli Studi della Tuscia, ITALY

## Abstract

This paper presents a novel optimization technique for energy management studies of an isolated microgrid. The system is supplied by various Distributed Energy Resources (DERs), Diesel Generator (DG), a Wind Turbine Generator (WTG), Photovoltaic (PV) arrays and supported by fuel cell/electrolyzer Hydrogen storage system for short term storage. Multi-objective optimization is used through non-dominated sorting genetic algorithm to suit the load requirements under the given constraints. A novel multi-objective flower pollination algorithm is utilized to check the results. The Pros and cons of the two optimization techniques are compared and evaluated. An isolated microgrid is modelled using MATLAB software package, dispatch of active/reactive power, optimal load flow analysis with slack bus selection are carried out to be able to minimize fuel cost and line losses under realistic constraints. The performance of the system is studied and analyzed during both summer and winter conditions and three case studies are presented for each condition. The modified IEEE 15 bus system is used to validate the proposed algorithm.

## Introduction

The higher penetration of renewable energy resources into the electrical grid, the use of distributed generation for large loads such as the industrial sector, the deregulation of electricity market and the introduction of private sector in an open market environment; all attracted the interest of researchers towards microgrids as the main composing element of the smart grid.

A micro grid is a small-scale network to supply several types of demands including electricity, heating, and cooling for a small community [1]. It is essentially an active distribution network, working either in grid connected or isolated mode. It is composed of distributed energy resources (DERs) at distribution level, and these resources are usually non-conventional. To investigate the optimization of a microgrid’s performance, the different components of the microgrid have to be modelled and simulated separately. These components are then integrated into a complete model of the microgrid. This is faced by challenges such as the problem of the absence of a physical node with large generation capability (slack bus) that is required for the load flow analysis. Another challenge is the dispatch of reactive power. Moreover, to ensure reliable and continuous operation to loads in a microgrid, an energy management system needs to control the operation of energy storage devices. These devices take advantage of the uncertainty of renewable DERs. Hydrogen energy storage has emerged recently in the form of an electrolyzer/ fuel cell system [2].

Previous research in the field of microgrid optimization shows that AI techniques are best used for optimization and power management of the microgrid [3]. GA is occasionally preferred in such applications, as the robustness of the algorithm in handling complex problems compensates for the computation time required to produce optimal solutions [4]. Newly developed techniques still require further research such as flower pollination algorithm, a nature inspired algorithm developed in [5,6] and shows great promise compared to older optimization algorithms.

Economic modelling of fuel cell and electrolyzer systems for microgrid optimizations has been studied in literature [7–10]. Moreover, issues like system sizing and optimization constraints were also presented. However, in [11, 12] it was concluded that Hydrogen storage systems are currently inefficient/ unfeasible. Such a system has a limited efficiency of 30% and is clearly dominated by traditional battery storage. However, due to its large storage capacity and lack of pollution bi-products it is expected that the research in the area of Hydrogen energy will provide solutions for efficient and feasible energy storage.

In the present paper, the modelling of different DERs in the microgrid is presented. Energy storage system by fuel cell/electrolyzer is studied and modelled. Different components are then integrated. In order to perform load flow analysis and active/reactive power dispatch the addition of reactive power as a decision variable in the optimization problem is not widely addressed; this allows the system to operate in a cost efficient manner with no need to address the reactive power with separate control systems. Slack bus selection is required due to the absence of a physical slack bus, as seen in previous literature [13, 14], the absence of a physical slack bus requires solutions specific for isolated microgrids. However, in our case, the selection of an optimal slack bus by the optimization algorithm enables the use of traditional solutions. Multi-objective constrained optimization is performed by non-dominated sorting genetic algorithm two (NSGA-II) and our novel Non-dominated Sorting Multi Objective (MO) optimization algorithm based on the newly developed flower pollination algorithm (FPA), which was not used to this kind of problem before, although it was used for relevant problems in the field as in [15–18]. Moreover, the algorithm itself was developed using non-dominated sorting for multi objective optimization, other techniques were used in previous literature as in [19–20]. The performance of the system is studied and analyzed during both summer and winter conditions and three case studied are presented for each condition. The performance of the novel MO optimization algorithm is analyzed and shows higher efficiency compared to the traditional NSGA-II algorithm.

## System modelling

In order to study the performance of a microgrid, different elements of the grid must be modelled. The microgrid under study is composed of a number of buses where on each bus there is either a load, photovoltaic array, wind turbine or diesel generators, similar to a real island, such a microgrid can operate in a stand-alone isolated mode or a grid connected mode. In addition to load and generation, Fuel cell/electrolyzer systems where used to provide storage using Hydrogen.

The following subsections demonstrate the modelling of different microgrid components and the power flow analysis.

### Diesel generators

The modularity of diesel generators makes them a suitable solution for isolated microgrids with more generators can be added when loads grow. Moreover, installation of diesel generators is easy as it comes usually in an enclosed set with all required control, protection and fuel systems. The Fuel cost function of a diesel generator can be expressed as [21]:

$$C_{F}=a_{i}+b_{i}P_{G}+c_{i}P_{G}^{2}$$ (1)

Where;

C_F_: fuel cost of generator i.
a, b, c: cost coefficients.
P_G_: power output of generator i.

### Photovoltaic modules

PVs attract a great deal of investments and research as they have major advantages such as short lead time to design, install, and start up a new plant. They are highly modular, hence, the plant economy is not a strong function of size. Power output matches very well with peak load demands. Static structures, no moving parts, hence, no noise. High power capability per unit of weight, longer life with little maintenance because of no moving parts, and High mobile and portable because of light weight [22]. The model in [23] provides the maximum power point (MPP) output from a PV module at any given irradiation and temperature:

$$P_{mppt}=V_{mpp}*I_{mpp}$$ (2)

Where:

P _mppt_: power output of a PV module.
V_mpp_: terminal voltage at MPP.
I _mpp_: module current at MPP.

The model reads environmental data such as temperature and irradiance. Data used in this research is taken from actual values recorded in the city of Hurghada, Egypt. The output of the model is the power produced by the PV plant.

### Wind turbines

The power extracted from a wind turbine can be expressed by [9]:

$$P_{wind}=\frac{1}{2}\rho *A*v^{3}*C_{P}$$ (3)

Where:

A: swept area of the turbine.
*ρ*: air density.
v: wind speed.
C_p_: Bitz constant.

This is valid only when wind speed lies between the cut in speed and the rated speed of the turbine. If wind speed is higher than rated but lower than cut out speed, the output remains constant at the rated power. If wind speeds are lower than cut in speed or higher that cut out speeds the turbine is either pitched out of wind or stalled to stop the blades. The model is capable of simulating output power variations with wind speed based on actual datasheet parameters of the Polaris 100kW wind turbine.

### Hydrogen energy storage

For peak shaving purposes, the microgrid is equipped with a Hydrogen energy storage system that utilizes the energy produced by renewable DERs. The storage system consists of a fuel cell that converts the chemical energy stored in a fuel (Hydrogen) directly into electrical energy [24], and an electrolyzer, which is a device where the reverse reaction is used to produce Hydrogen from water by the process of electrolysis. The reversible chemical reaction between Hydrogen and Oxygen produces electrical energy, heat and water as illustrated by Eq (4).

$$2H_{2}+O_{2}->H_{2}O+elec.energy+heat$$ (4)

Generated Hydrogen is stored in tanks which allow the use of hydrogen for both short term storage and long term storage. Hydrogen storage has very high efficiency compared to pumped hydroelectric storage (large scale) or batteries (small scale). However, the poor efficiencies of electrolyzers and fuel cells reduce the overall system efficiency to experimental values of 30% [11]. Further research and developments in the manufacturing of FCs and electrolyzers make Hydrogen storage a promising solution to replace other storage techniques. The model of a Hydrogen storage system proposed in [12] is intended to calculate the amount of Hydrogen consumed by a fuel cell with respect to produced electrical power. In addition, the model does the reverse process for an electrolyzer. The model in [12] is expressed as:

$$E_{FC}=E_{H2}*\eta_{FC}$$ (5)

Where:

E_FC_: power produced by the Fuel cell.
E_H2_: Hydrogen consumed by the fuel cell.
η_FC_: fuel cell efficiency.

$$E_{H2}=E_{elyz}*\eta_{elyz}$$ (6)

Where:

E_elyz_: power consumed by the electrolyzer.
E_H2_: Hydrogen produced by the electrolyzer.
Η_elyz_: electrolyzer efficiency.

In case of using a Hybrid system with energy storage (conventional DG, renewable DERs, and Hydrogen energy storage), the system is capable of peak shaving in a manner that the system will calculate the peak time consumption during night hours. During day hours, the microgrid will use power from DERs to supply peak demand and store the required amount of Hydrogen. During night, stored Hydrogen will be used to supply peak demand. Thus, enabling the conventional DGs to operate at lower power outputs with fewer oscillations.

The energy required during night peak time is estimated using Eq (7) and the hourly power required by Fuel cell to store sufficient amount of Hydrogen is as in Eq (8).

$$E_{night}=\sum_{i=1}^{Hr_{night}}{P_{i}^{peak}}_{i}-P_{i}^{base}$$ (7)

Where:

E _night_: energy consumed at night peak load hours.
$P_{i}^{peak}$: Peak load at hour i.
$P_{i}^{base}$: Base load at hour i.
Hr_night_: Number of night hours.

$$P_{storage}=\frac{E\_night}{PSH*\eta_{fc}*\eta_{elyz}}*SF$$ (8)

Where:

P _storage:_ power storage required for night operations.
PSH: peak sun hours.
SF: safety factor (slightly more energy is stored than required to avoid unexpected variations in weather and night loads).

### Power flow analysis

For an islanded microgrid, the absence of a node with large generation capability (slack bus) is a problem. Slack bus, in traditional load flow analysis, is necessary to achieve load matching, and it is usually a dispatchable generation unit with large capacity (theoretically infinity). However, in an islanded microgrid that relies on micro sources and DERs, such bus does not exist and traditional load flow analyses are not applicable. The most promising solution to this problem is to include slack bus selection as part of the optimization algorithm (variable slack bus) such that the algorithm will select the slack bus to achieve optimum operation under the given objectives and constraints. As a result, the solution vector of the optimization problem must include a variable that represents slack bus selection. Although making the process harder, the selection of slack bus will affect the load flow analysis and change values of line losses and bus voltages, further improving the overall economic and technical performance of the system.

## Optimization problem

In islanded microgrids, certain aspects must be considered in the operation of the microgrid. For instance, generation and storage must be planned according to load demand and long term energy balance. However, Short term scheduling must be done in order to provide higher reliability and energy quality. Economic operation must be achieved at all times through energy management and if required; demand side management. Issues such as reactive power compensation and power electronic interfacing between different components must be considered. Optimization of generation and optimal power flow is vital in case of islanded microgrids, and this is carefully buckled in this research.

For the microgrid previously modelled in section II, it is required to optimize the performance by minimizing both fuel cost (f_1_) and line losses (f_2_).

$$f_{1}=\sum_{i=1}^{N_{G}}C_{G}(i),f_{2}=\sum_{i=1}^{N_{line}}I_{line}^{2}(i)*R(i)$$ (9)

The optimization problem requires the use of a multi objective optimization algorithm that is capable of handling both equality constraints (such as active and reactive load matching) and inequality constraints (such as minimum and maximum generation limits, Hydrogen tank capacity and bus voltage magnitudes). These constraints are illustrated in Eqs (10–19). Eqs 10 and 11 are the active (P)/reactive (Q) power matching constraints where subscript *g* is for generation, *D* for demand, and *l* for losses.

$$\sum_{i=1}^{N_{G}}P_{g}(i)=\sum_{i=1}^{N_{bus}}P_{D}(i)+\sum_{i=1}^{N_{line}}P_{l}(i)$$ (10)

$$\sum_{i=1}^{N_{G}}Q_{g}(i)=\sum_{i=1}^{N_{bus}}Q_{D}(i)+\sum_{i=1}^{N_{line}}Q_{l}(i)$$ (11)

Eq 12 represents the voltage constraints at each bus *i*.

$$V_{min}<V_{bus}(i)<V_{max}$$ (12)

Eqs (13–18) are the constraints for the power consumption/generation of each component in the system.

$$P_{g_{min}}(i)<P_{g}(i)<P_{g_{max}}(i)$$ (13)

$$Q_{g_{min}}(i)<Q_{g}(i)<Q_{g_{max}}(i)$$ (14)

$$S_{{PV}_{min}}<S_{PV}<S_{{PV}_{max}}$$ (15)

$$S_{{wind}_{min}}<S_{wind}<S_{{wind}_{max}}$$ (16)

$$S_{{FC}_{min}}<S_{FC}<S_{{FC}_{max}}$$ (17)

$$P_{{elyz}_{min}}<P_{elyz}<P_{{elyz}_{max}}$$ (18)

Eq 19 is the constraint for the Hydrogen storage tank capacity.

$$H_{{tank}_{min}}<H_{tank}<H_{{tank}_{max}}$$ (19)

The solution to the optimization problem is a vector composed of the active/reactive power output of each generation unit (continuous) and the slack bus number (discrete). In this case, the algorithm is a mixed encoding GA. and the solution vector is x where:

$$x=[P_{1}\;P_{2}\ldots ..Q_{1}\;Q_{2}\ldots ..SBID]$$ (20)

Where:

P1-5: active power output of a generator.
Q1-5: reactive power output of a generator.
SBID: slack bus selection.

### Non-dominated sorting genetic algorithm II

In order to solve such a problem, NSGA-II was used. Genetic algorithm finds pareto-front of non-dominated solutions to a multi objective optimization problem by initializing a random population of solution vectors (chromosome) where solution holds the set of decision variables (genes) and the fitness values. After the initialization step, chromosomes are selected for mating based on a tournament, where better chromosomes have a better chance for mating (similar to genetic evolution), mating between chromosomes is done using crossover, where an offspring (a new solution) has some features from each parent, or mutation, where a single parent produces a single offspring by mutation genes. The new population (offspring) is then merged with the original population and the resulting population is sorted based on dominance relations [4]. As a variant of GA, NSGA-II features unique constraint handling ability by assigning very high cost and losses values to any solution that violates one or more of the constraints. The algorithm also features elitism, by missing the original population and the offspring, best solutions across both populations are selected, keeping the best solutions intact across generations [4].

### Proposed algorithm: Modified flower pollination algorithm

The newly developed flower pollination algorithm (developed in 2012 by Yang [5]) is inspired from the process of pollination of flowers by birds or insects (global pollination) or by spread of pollens (local Pollination). The process starts by generation of random population of flowers, the fit flower is selected and pollens are then spread according to global and local pollination models such that [5]:

$$x_{i}^{t+1}=x_{i}^{t}+\gamma L(x_{i}^{t}-g_{*})$$ (21)

$$L∼\frac{\lambda \Gamma (\lambda )sin(\frac{\pi \lambda }{2})}{\pi }*\frac{1}{s^{1+\lambda }}$$ (22)

The resulting population of flowers is evaluated and compared to the best flower. This process is repeated until stopping condition is reached.

The original algorithm was only usable for single objective unconstrained problems. The algorithm was improved in 2013 to handle multi-objective optimization problems by converting the problem to a single objective problem by means of a weighted sum [6]. For purposes of microgrid optimization in this paper, the algorithm was developed to handle multi-objective problems by non-dominated sorting, the proposed algorithm features elitism and constraint handling in a manner similar to NSGA-II. The proposed algorithm showed promising results and an improvement in overall microgrid performance and convergence time.

## Simulation & results

**Fig 1:**
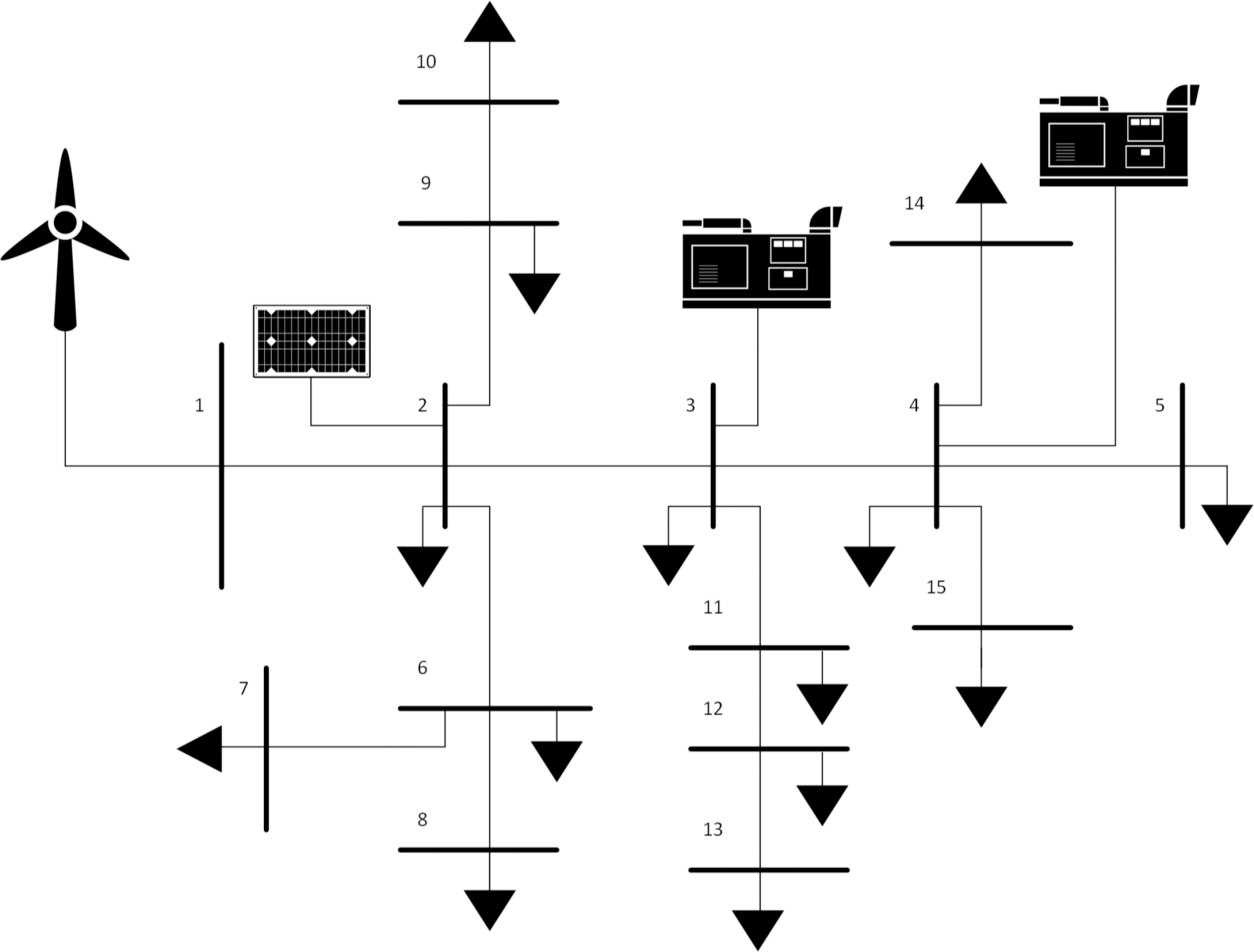
Modified IEEE 15-bus system.

In order to test the algorithm on a microgrid, an IEEE-15 bus system used in [25] is modified to utilize renewable DERs along with conventional diesel generators and energy storage system. The modified microgrid is shown in Fig 1. Three different configurations are simulated; these are:

1. System is supplied only by diesel generators.
2. A hybrid system supported by renewable DERs.
3. A hybrid system supported by renewable DERs and energy storage system.

**Fig 2:**
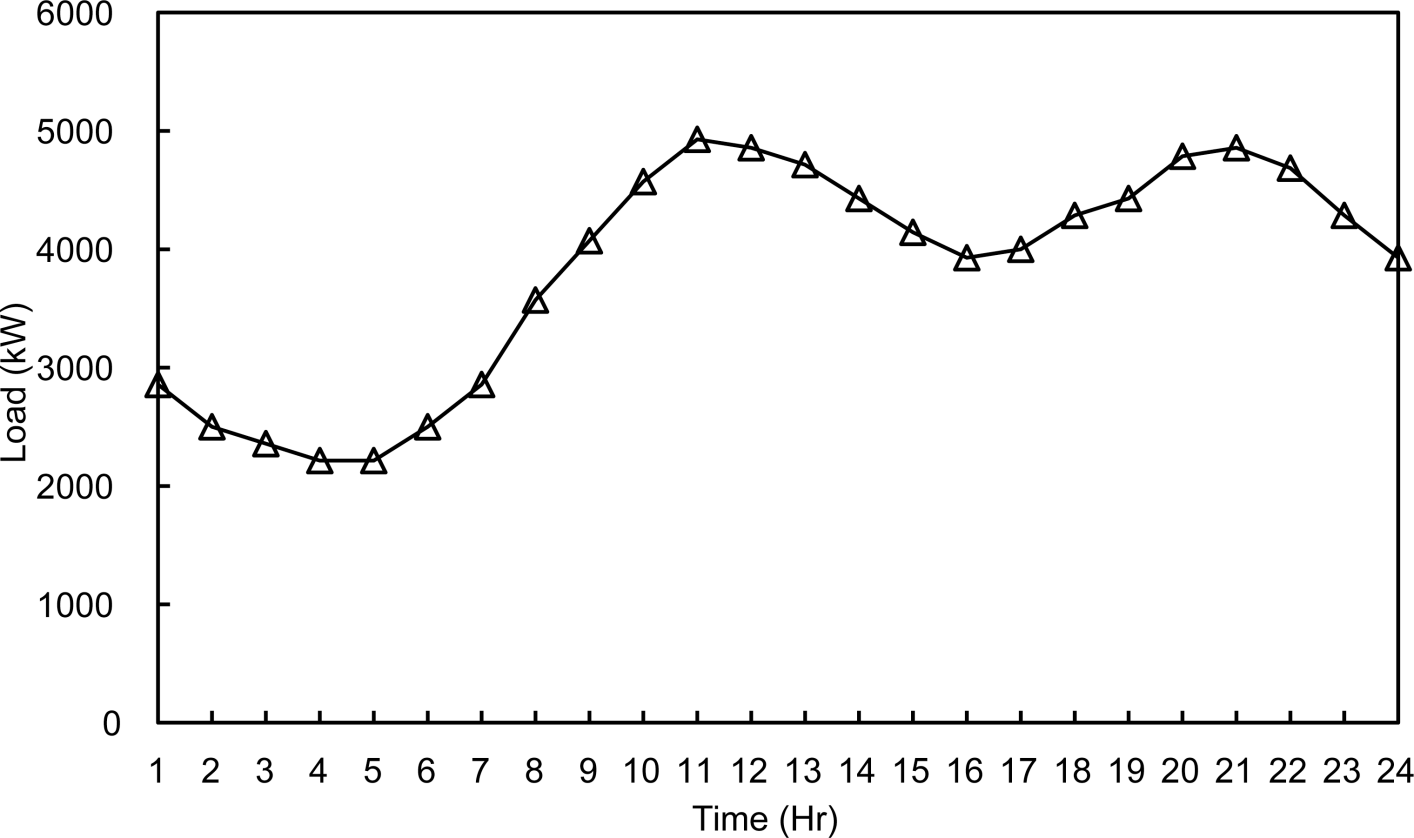
Summer day load profile.

**Fig 3:**
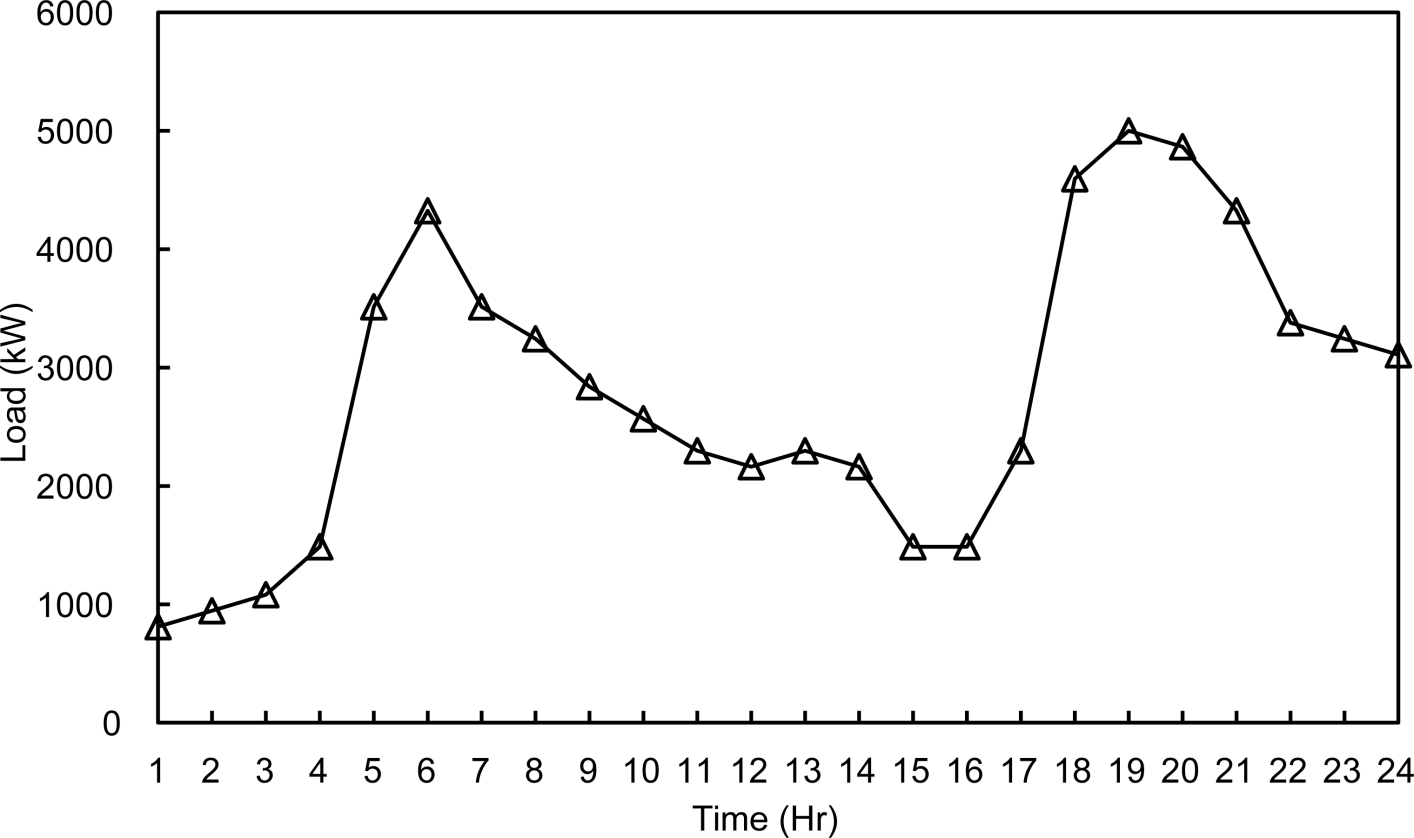
Winter day load profile.

The three configurations were tested during a summer day and a winter day to simulate different load and environmental conditions as in S1 and S2 Files. The two optimization algorithms are compared. The microgrid has a peak load of 3.5 MWatts, distributed on 15 buses with power factor of 0.7. In order to simulate all possible cases of the microgrid, two daily load profiles shown in Figs 2 and 3 are used in the simulation.

**Fig 4:**
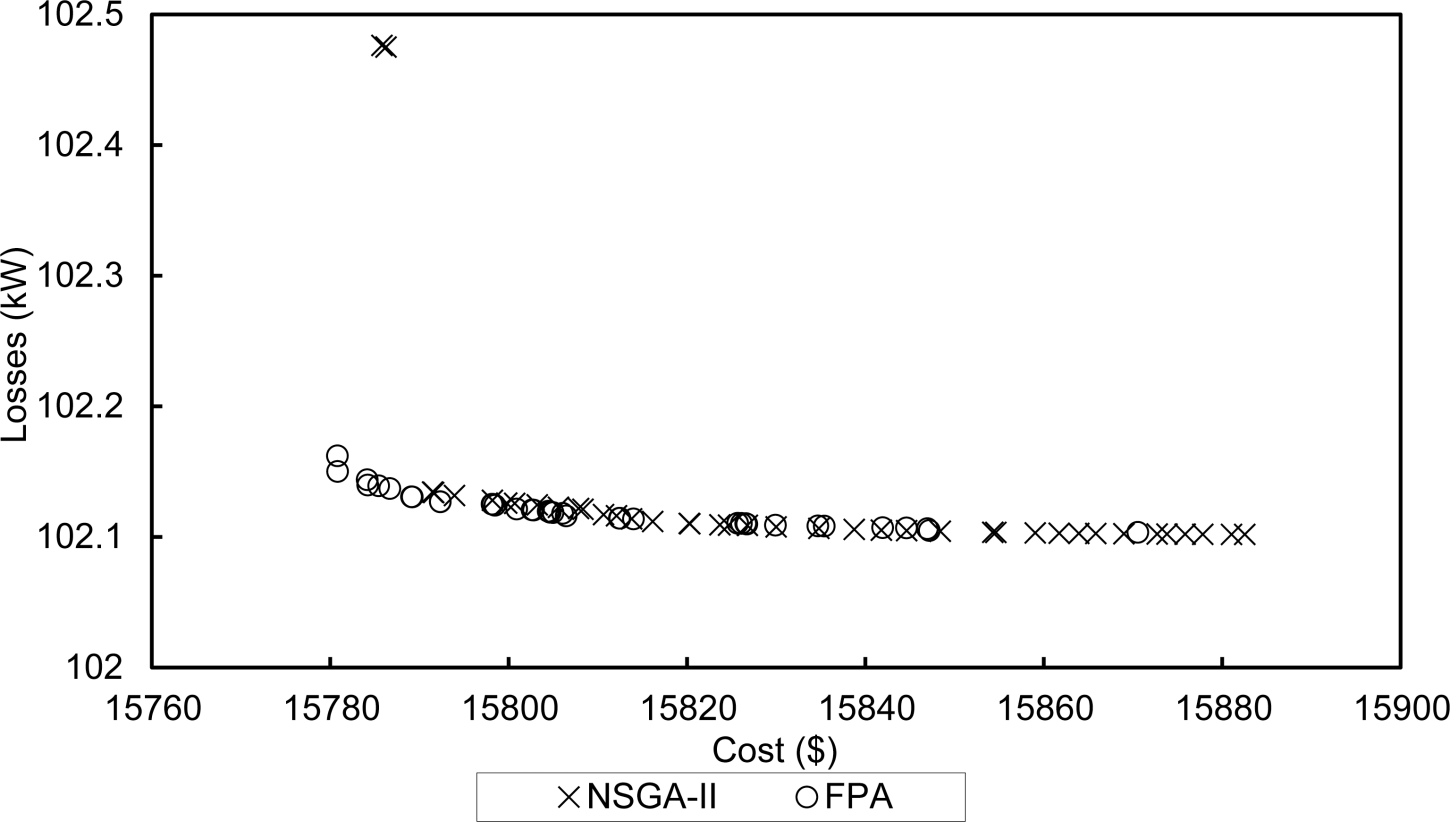
Pareto-fronts of NSGA-II and FPA.

The components of the system are modelled to calculate the output power contributed from renewable DERs according to environmental conditions. The output of a PV power plant is dependent on solar irradiation and temperature. Whereas, a wind turbine will produce an output depending on wind speeds and varies throughout a day. The available power from renewables is compared with the load demand. The algorithm determines the operating mode of the microgrid. Whether the system will use diesel generators to match the load demand, or in case of using a storage system, use surplus power to store Hydrogen and use Hydrogen to supply power shortages. Based on the operating mode of the microgrid, the optimization algorithm will determine the optimum active/reactive output of diesel generators. Moreover, the algorithm will select the optimum slack bus in order to achieve minimum fuel cost and line losses. From the pareto-front obtained by the optimization algorithm, we take the solution with the lowest fuel cost as the set point for generators. By doing this, we make a slight compromise in losses in exchange for lower costs. This can be tolerated since the power lost is already accounted for in the generation cost function. For example, Fig 4 shows the pareto-front obtained by NSGA-II and our proposed FPA for a specific hour. It can be seen that the solution with the lowest fuel cost found by FPA (15780.8, 102.162) dominates that found by NSGA-II (15785, 102.476).

### Configuration 1: Diesel generators only

In this case study, the microgrid is supplied by diesel generators only as shown in Table 1. Since there is no other source of energy, diesel generators must be able to supply the peak load plus losses.

**Table 1:** Generation data for configuration 1.

Bus number	DER type	Minimum operating limits	Maximum operating limits
3	5*Diesel Generators (Pac1)	Pmin = 0 Qmin = 0	Pmax = 400kW Qmax = 500kVAR
4	5*Diesel Generators (Pac2)	Pmin = 0 Qmin = 0	Pmax = 400kW Qmax = 500kVAR

In this case study the algorithm will evaluate the hourly load demand depending on the daily load profiles shown in Figs 2 and 3. Then, the optimization algorithm will find the optimal active/reactive dispatch for each of the diesel generators according to the optimization objectives and constraints.

**Fig 5:**
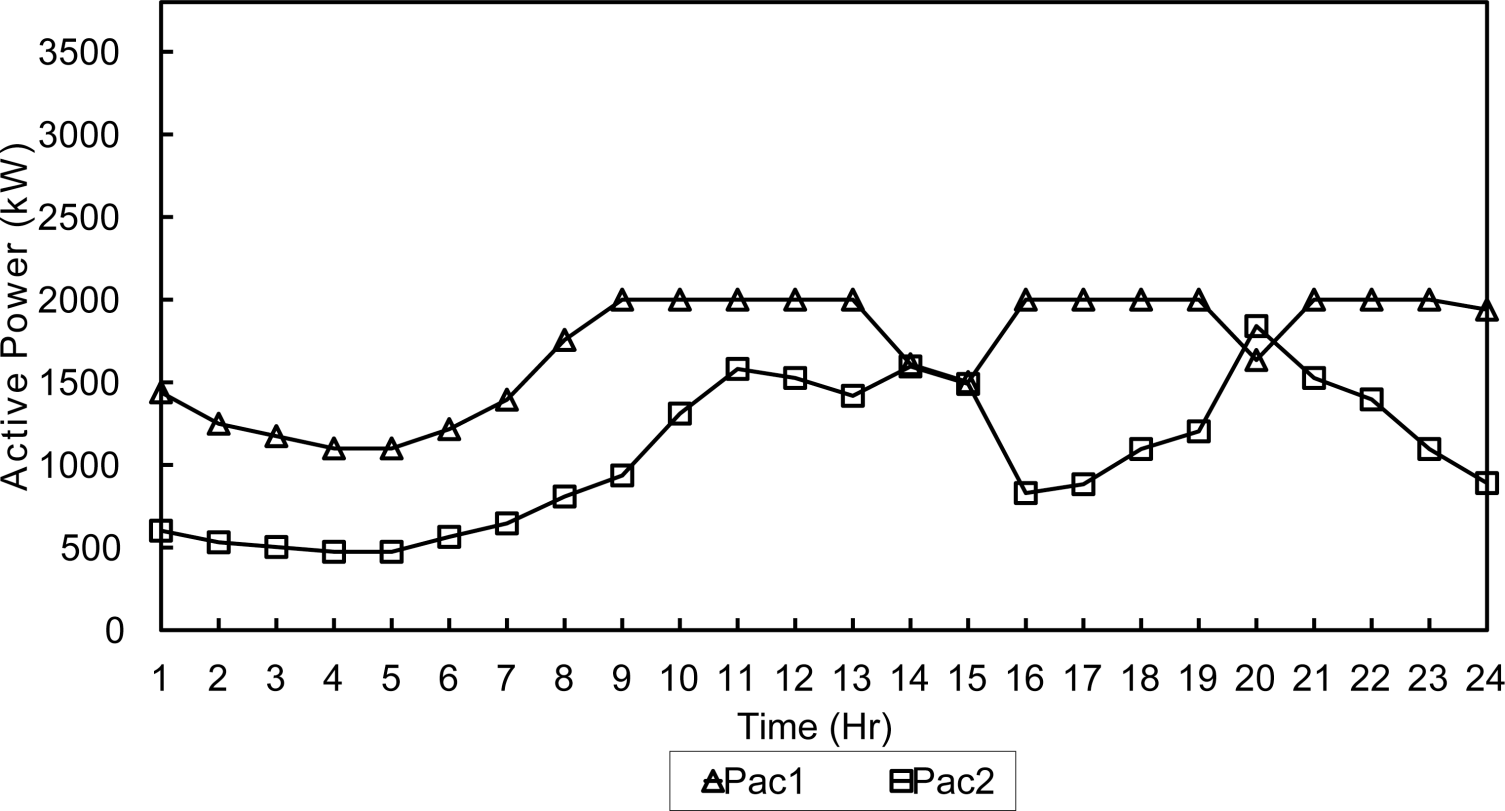
Active dispatch of generators-NSGA-Summer.

**Fig 6:**
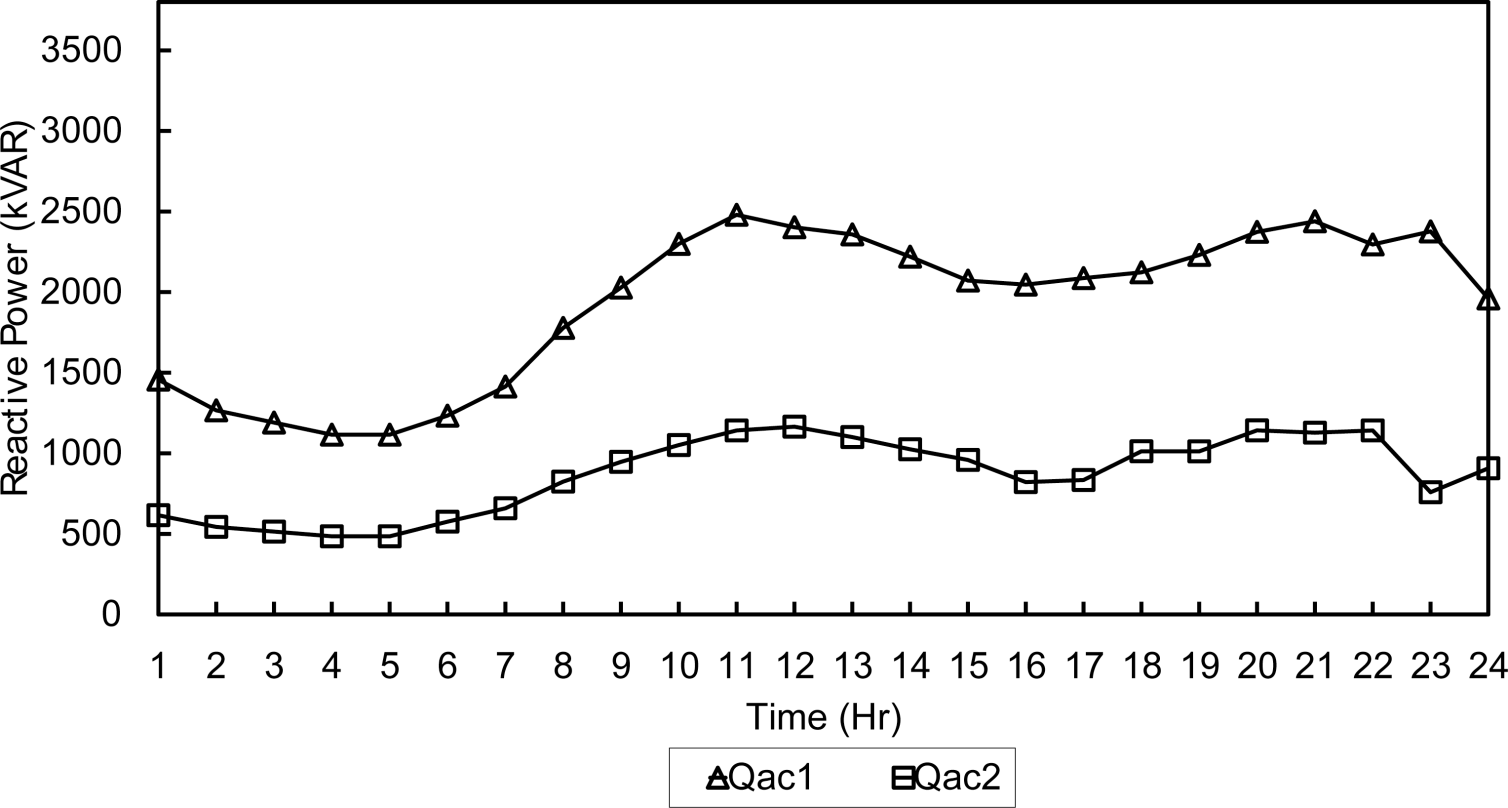
Reactive dispatch of generators-NSGA-Summer.

Moreover, the algorithm will select the optimum slack bus among the two buses 3 and 4. This will have direct effect on line losses and subsequently, on generation costs. Both optimization algorithms were tested using a population of 40 solutions over 500 generations. For a summer day, NSGA-II obtained a daily fuel cost of 784,723$ and total line losses of 1960.9kWh. The output of each generator set is shown in Figs 5 and 6.

When the same scenario was optimized using the proposed FPA algorithm, the modified flower pollination algorithm appears to obtain better results over the day. The total fuel costs in this scenario was 739,021$ with a 5.8% reduction compared to NSGA-II. The total line losses were 1986kWh. Thus, total line losses were increased by 1.28%. The lower cost is achieved by better load sharing among the generator sets.

When the same configuration was tested over a winter day, NSGA-II obtained fuel cost of 470,944$ daily and total line losses of 1158.8kWh were lost.

The modified FPA obtained better results as the system had total fuel cost of 451,007$ with a reduction of 4.2% compared to NSGA-II. Line losses were also reduced by 1.49% to become 1168.14kWh daily.

### Configuration 2: Hybrid system

In this case study, the algorithm will determine the power available from renewable DERs (PV, WT). This energy will be used to supply the active load demand. Diesel generators are then dispatched to supply the remaining active power, line losses, and the reactive power demand of the system. The installed DERs and generators are shown in Table 2.

Due to the intermittent nature of renewable DERs, installed diesel generators must be sufficient to supply the peak load at any time.

**Table 2:** Generation data for configuration 2.

Bus number	DER type	Minimum operating limits	Maximum operating limits
1	10*Wind Turbines	0	100kW
2	PV power plant	0	1000kW
3	5*Diesel Generators (Pac1)	Pmin = 0 Qmin = 0	Pmax = 400kW Qmax = 500kVAR
4	5*Diesel Generators (Pac2)	Pmin = 0 Qmin = 0	Pmax = 400kW Qmax = 500kVAR

The addition of renewable DERs removed the day peaks. The microgrid consumed daily fuel cost of 518,001$ and line losses were reduced to 1745.4kWh. When the same scenario was simulated using modified MOFPA, the daily fuel cost was reduced by 4.14% to become 596,548$. Line losses, however, increased to 1753.76 with an increase of 0.47%.

For a winter day, output from PVs is noticeably less compared to a summer day. However, higher WT output is expected due to windy conditions. When NSGA-II is used to optimize the performance of the microgrid, the active dispatch of diesel generators varies greatly. This is due to the variation of the winter load profile. Also, due to the time difference between peak demand times and peak PV time. During peak sun hours, PVs produce their maximum output and the demand is very low; this results in a very low output from generator set 1 (Bus 3). Bus 4 is preferred in this case as the contribution of DERs at buses 1, 2 will reduce the total line losses.

The microgrid had daily fuel cost of 364,853$ which is a noticeable reduction compared to the diesel only case study. Daily line losses were reduced to 1095.93kWh.

**Fig 7:**
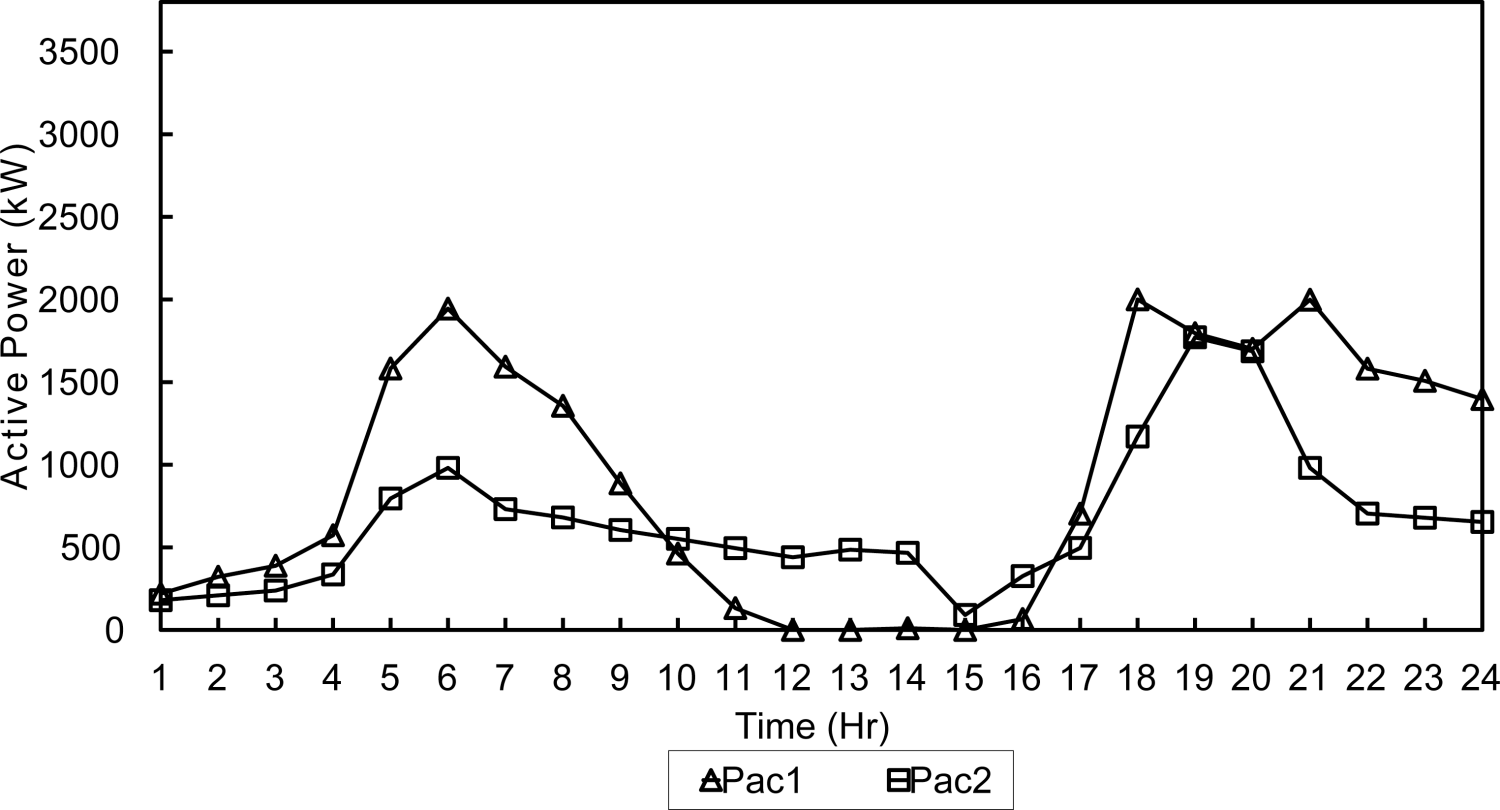
Active dispatch of generators-NSGA-Winter.

**Fig 8:**
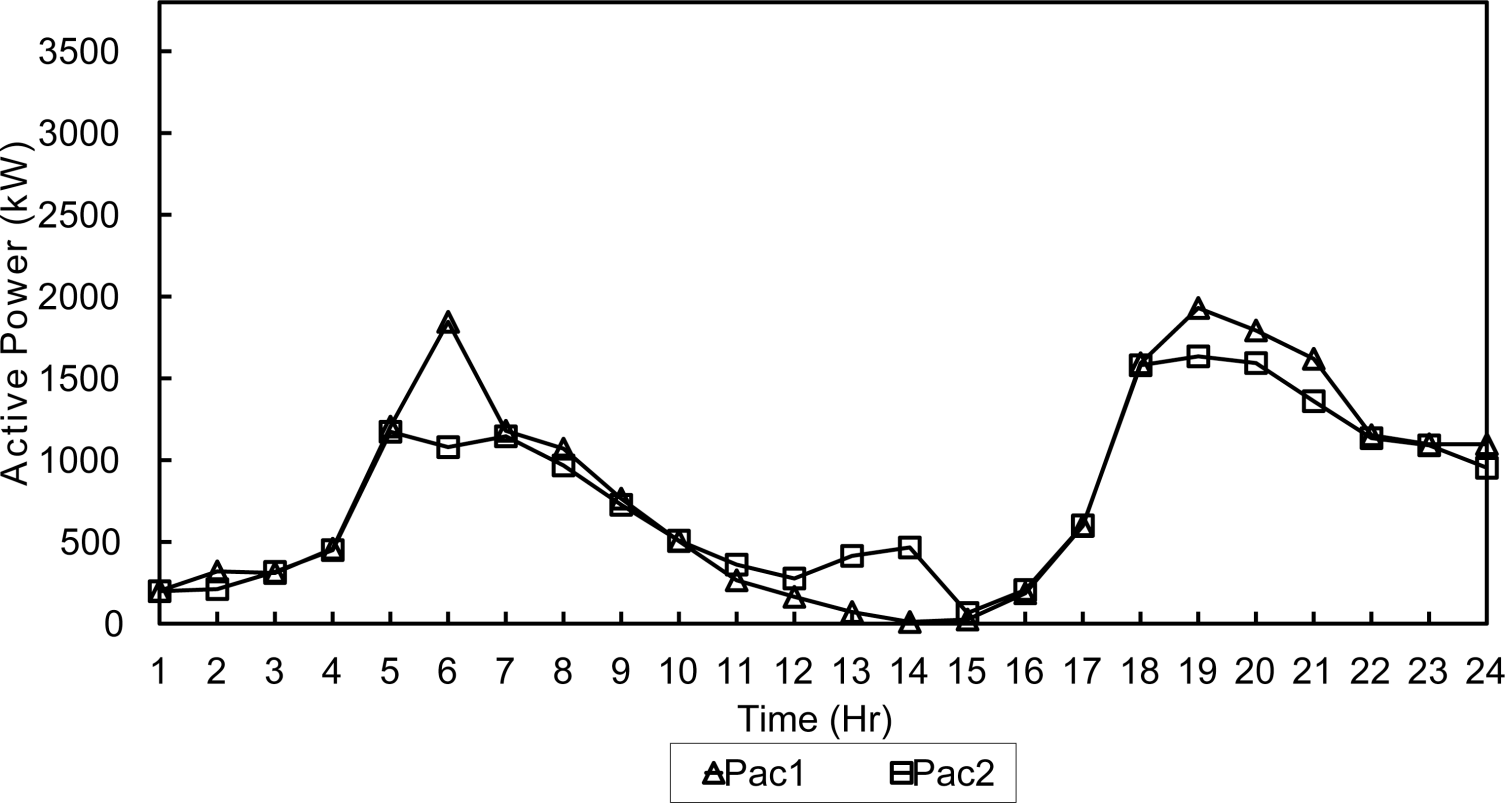
Active dispatch of generators-FPA-Winter.

When the same scenario is optimized using proposed FPA, daily fuel cost was reduced by 6.4% to reach 341,381$. Line losses increased slightly to become 1102.7kWh (0.61% increase) when compared to the same scenario simulated by NSGA-II. Fluctuations in power output of diesel generators can be noticed in winter day, and are less extreme when using FPA as shown in Figs 7 and 8.

### Hybrid system with hydrogen storage

In this case study, Hydrogen storage system is added to the microgrid. The microgrid now has a peak shaving feature, which means that during day, renewable DERs will be responsible for supplying the peak demand, while in night time; peak demand will be supplied by fuel cells using the energy stored during day.

The algorithm will calculate the energy required during night time peaks. Divided by the storage system cumulative efficiency and the peak sun hours, the energy required for storage every hour will be evaluated. When this energy is available from renewables, it will be stored. However, during day time peaks priority will be given to supplying the load demand. This operation is expected to reduce the fluctuations in diesel generator output seen in the previous cases. Also fuel costs and line losses are expected to drop. However, it should be noted that Hydrogen energy storage systems are economically infeasible in the current time due to the high cost/ low efficiency of hydrogen energy systems i.e. fuel cells, electrolyzers.

Moreover, due to the presence of storage devices, downsizing of diesel generators can be considered as they are not required to supply the full load but only a base load. The microgrid was supplied by DERs shown in Table 3. The specifications of the hydrogen energy storage system are show in Table 4. The base load is at 2500kW and Hydrogen has a lower heating value of 33.33kWh/kgH2. The electrolyzer must be able to withstand all the power output from PVs, while FC only needs to cover the peak demand of 1000kW.

**Table 3:** Generation data for configuration 3.

Bus number	DER type	Minimum operating limits	Maximum operating limits
1	10*Wind Turbines	0	100kW
2	PV power plant	0	3500kW
3	4*Diesel Generators (Pac1)	Pmin = 0 Qmin = 0	Pmax = 400kW Qmax = 500kVAR
4	4*Diesel Generators (Pac2)	Pmin = 0 Qmin = 0	Pmax = 400kW Qmax = 500kVAR

**Table 4:** Hydrogen storage system specifications.

Bus number	Device	Minimum operating limits	Maximum operating limits	Specifications
2	Fuel cell array	0kW	1000kW	*η*_*FC*_ = 38.36%
2	Electrolyzer array	0kW	3500kW	*η*_*elyz*_ = 34.35%
-	Hydrogen tank	0 kg	300kg	-

The Energy consumed by the electrolyzer is much higher than energy produced by the fuel cell array. This is caused by the low efficiency of the Hydrogen energy storage system. Currently, Hydrogen energy systems are characterized by low efficiencies which make them infeasible in most cases. However, the situation is expected to change with the ongoing research and improvements in this field.

The addition of an energy storage system reduced fuel costs and line losses to 455,670$ and 1717.12kWh daily. Better results can be achieved with improvements in the electrolyzer/Fuel cell efficiency. When, FPA was used to optimize the performance of the same system, a 2.5% reduction in fuel cost was obtained. On the other hand, losses were increased by 0.23%.

The same system was tested during a winter day, the output from PVs was decreased due to less irradiation, and output from WTs increased slightly due to higher wind speeds. Due to less output from PV, and less energy requirements through the day, the stored amount of Hydrogen determined by the algorithm was less than that of a summer day, and electrolyzer/FC power was less.

However, in case of using the proposed FPA, better load sharing among the generators is achieved, fuel cost is reduced by 4.2% compared to NSGA-II and line losses increased by 0.1%.

**Fig 9:**
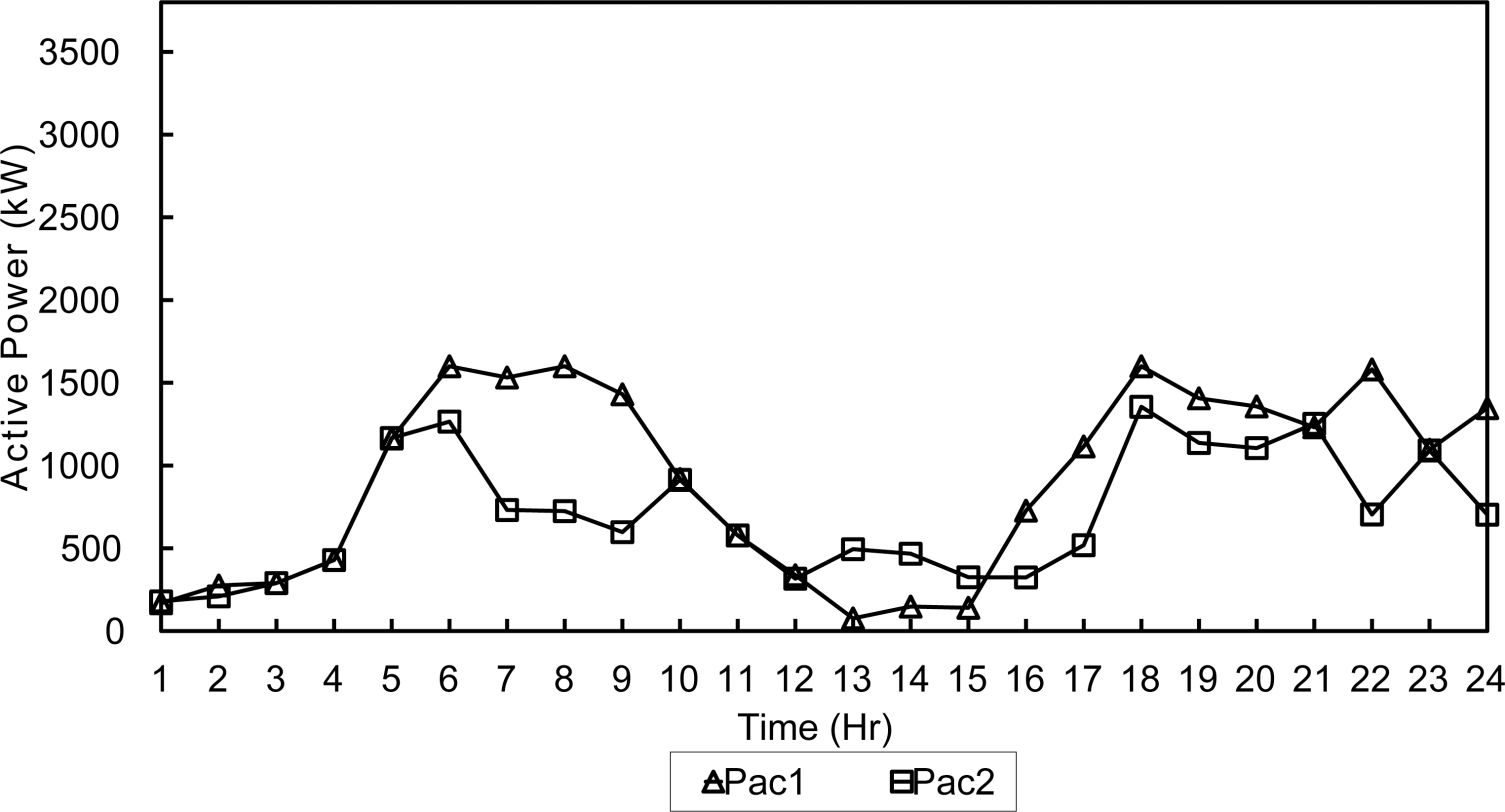
Active dispatch of generators-NSGA-Winter.

**Fig 10:**
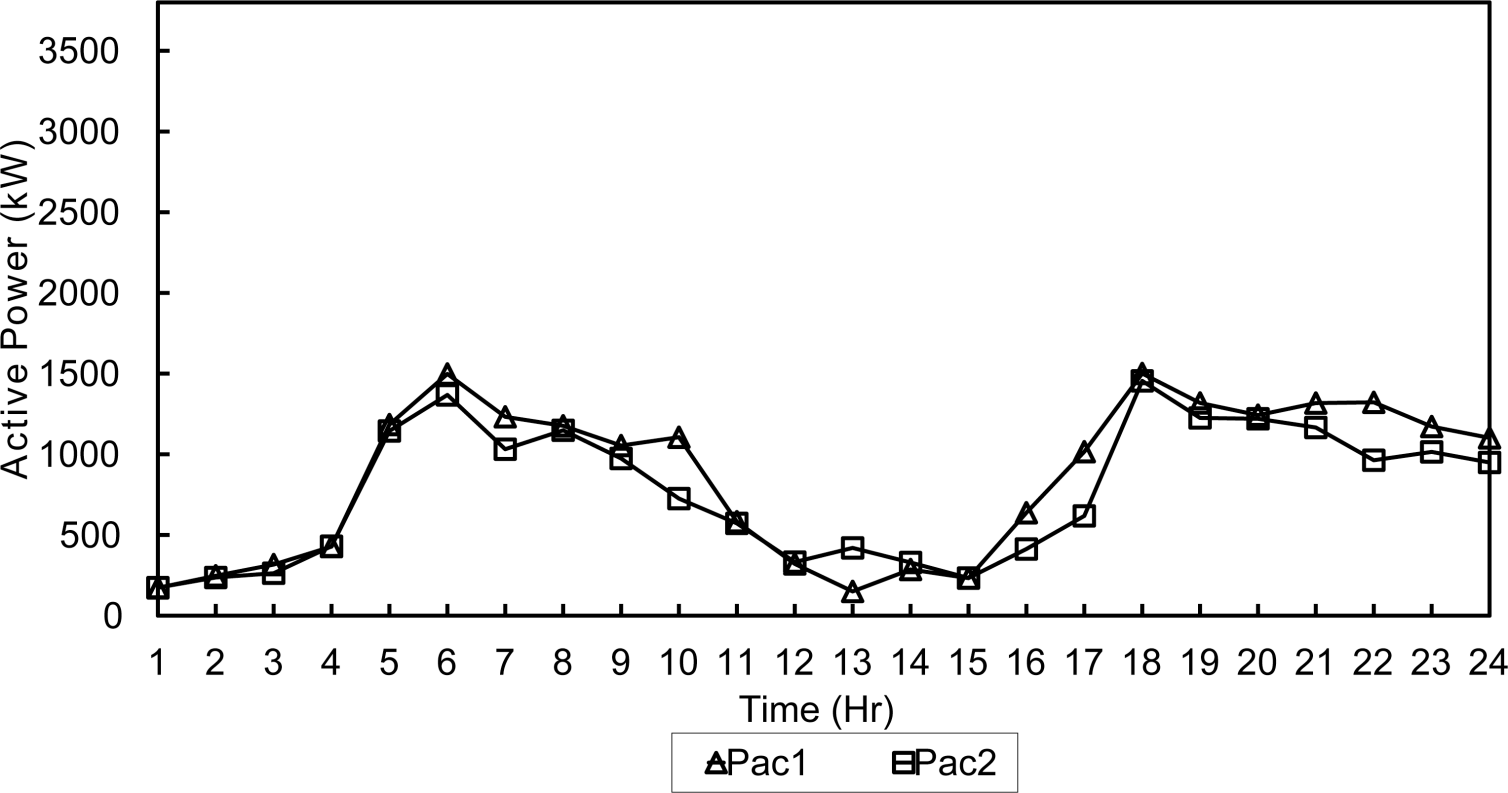
Active dispatch of generators-FPA-Winter.

The application of the peak shaving technique resulted in less fluctuations in the output of diesel generator sets. This can be seen in Figs 9 and 10 where the active dispatch of diesel generators is shown.

## Summary & conclusions

From the case studies and simulation scenarios conducted, various observations can be made. A comparison can be made between different options of generations; different optimization algorithms can be made.

From the previous illustrated results, it can be concluded that the addition of renewable DERs achieved lower fuel costs and less line losses. This could provide cheaper energy for consumers of a microgrid, and moreover, higher energy efficiency is obtained.

The addition of Hydrogen storage system was beneficial that it reduced fuel costs, improved the form factor of diesel generator sets, and introduced the option of downsizing the generators.

The improvements in microgrid performance were more noticeable during summer days when the demands are higher and the output from PVs is high and has high degree of certainty. Table 5 shows a comparison between the different options of supplying the microgrid for a summer day.

**Table 5:** Summer day results comparison.

Case	Parameter	NSGA-II	MOFPA
Diesel only	Fuel cost ($)	784722.8	739021.4
	Losses (kWh)	1960.944	1985.983
	Average voltage (Pu)	0.977529	0.97778
Hybrid	Fuel cost ($)	518001.3	496548.2
	Losses (kWh)	1745.399	1753.757
	Average voltage (Pu)	0.980114	0.980069
Hybrid with storage	Fuel cost ($)	455669.7	444320.9
	Losses (kWh)	1717.116	1721.197
	Average voltage (Pu)	0.980488	0.980516

The comparison between the traditional NSGA-II and the proposed modified FPA is, in fact, in favour of the proposed algorithm. When using the same optimization parameters, the proposed algorithm obtained better fuel costs in all cases compared to NSGA-II. The improvements range from 2% to 6.5% in some cases, with a compromise of very small ratios in the second objective (line losses). This can be tolerated as losses already are taken into account in the cost function (as part of the power generated), which means that the system runs with higher losses but in a more cost/fuel efficient manner due to better utilization of DGs.

In conclusion, an isolated microgrid was modelled accommodating different types of DERs, and a hydrogen storage system consisting of an electrolyzer and a hydrogen tank. Two different optimization techniques were used; NSGA-II and a proposed modified MOFPA. Although MOFPA was not previously used in similar applications, however it showed a significant improvement in the results obtained.

Reactive power was rarely optimized in previous research. However, including reactive power as an optimization parameter helped to reduce line losses and improve the overall performance of the grid. Moreover, this research highlighted a common problem in isolated microgrid; the absence of slack bus to be used in order to analyze the power flow in the microgrid. Thus, the traditional NR power flow analysis was modified to include the slack bus as an optimization variable, solved by mixed encoding GA. An overview of the obtained results shows that the addition of renewable DERs reduced the systems fuel cost by a large percentage of 33% and decreased daily line losses by 11%. The further usage of energy storage techniques reduced the costs by 11% more and slightly reduced daily line losses. The total improvement due to the usage of a complete hybrid system was 41% in fuel costs and 12% in line losses. Hydrogen energy storage techniques although proved economically unfeasible in the present, require more research and improvements in order to improve the efficiencies and fixed costs of different elements.

The proposed modified MO FPA proves worthy of consideration as it obtained results better by 2–6% in most cases. Moreover, the algorithm proved to converge towards an optimum solution 10 times faster than NSGA-II in most cases.

The scenarios considered in this paper are used to present the capability of the proposed algorithm to handle cases of high demand (as in summer) or high rapid variations in the demand pattern (as in winter). Future work can include the study of different load patterns, and larger systems.

## Supporting information

S1 File Environmental data. (PDF)

S2 File Load profiles. (PDF)

## References

[1] M. Modiri-Delshad; N. A. Rahim, Optimal operation of microgrid systems, 3rd IET International Conference on Clean Energy and Technology (CEAT) 2014. pp. 1–6. 2014.

[2] S. Tamalouzt, N. Benyahia, T. Rekioua, D. Rekioua and R. Abdessemed, Wind turbine-DFIG/photovoltaic/fuel cell hybrid power sources system associated with hydrogen storage energy for micro-grid applications, 2015 3rd International Renewable and Sustainable Energy Conference (IRSEC), Marrakech, 2015, pp. 1–6.

[3] Narkhede, M.S.; Chatterji, S.; Ghosh, S., Trends and challenges in optimization techniques for operation and control of Microgrid—A review, 2012 1st International Conference on Power and Energy in NERIST (ICPEN), pp.1, 7, 28–29 Dec. 2012.

[4] Deb K, Pratap A, Agarwal S, Meyarivan T, A fast and elitist multiobjective genetic algorithm: NSGA-II, IEEE Trans. on Evolutionary Computation, vol.6, No.2, pp182–197, 2002.

[5] Yang X. S. (2012), Flower pollination algorithm for global optimization, in: Unconventional Computation and Natural Computation, Lecture Notes in Computer Science, Vol. 7445, pp. 240–249.

[6] Yang Xin-She, Karamanoglu Mehmet, He Xingshi, Multi-objective Flower Algorithm for Optimization, Procedia Computer Science, Volume 18, 2013, Pages 861–868, ISSN 1877-0509.

[7] Jianxin Xu; Sicong Tan; Panda, S.K., Optimization of economic load dispatch for a microgrid using evolutionary computation, IECON 2011—37th Annual Conference on IEEE Industrial Electronics Society, pp.3192,3197, 7–10 Nov. 2011.

[8] El-Sharkh M.Y., Tanrioven M., Rahman A., Alam M.S., Cost related sensitivity analysis for optimal operation of a grid-parallel PEM fuel cell power plant, Journal of Power Sources, Volume 161, Issue 2, 27 10 2006, Pages 1198–1207, ISSN 0378-7753.

[9] D. B. Nelson, M. H. Nehrir, and C. Wang, Unit sizing of stand-alone hybrid wind/PV/fuel cell power generation systems, in Proc. IEEE Power Eng. Soc. Gen. Meeting, 2005, pp. 2115–2122.

[10] El-Sharkh M.Y., Rahman A., Alam M.S., Evolutionary programming-based methodology for economical output power from PEM fuel cell for microgrid application, Journal of Power Sources, Volume 139, Issues 1–2, 4 1 2005, Pages 165–169, ISSN 0378-7753.

[11] Aculik, J.; Hradilek, Z.; Moldrik, P.; Minarik, D., Calculation of efficiency of hydrogen storage system at the fuel cells laboratory, Proceedings of the 2014 15th International Scientific Conference on Electric Power Engineering (EPE), pp.381,384, 12–14 May 2014.

[12] Dufo-López Odolfo, Bernal-Agustín José L., Contreras Javier, Optimization of control strategies for stand-alone renewable energy systems with hydrogen storage, Renewable Energy, Volume 32, Issue 7, 6 2007, Pages 1102–1126, ISSN 0960-1481.

[13] Esmaeli A., Abedini M., Moradi M.H. “A novel power flow analysis in an islanded renewable microgrid”, (2016) Renewable Energy, 96, pp. 914–927.

[14] Abedini M. “A novel algorithm for load flow analysis in island microgrids using an improved evolutionary algorithm”, (2016) International Transactions on Electrical Energy Systems, 26 (12), pp. 2727–2743.

[15] Shilaja C., Ravi K. “Optimization of emission/economic dispatch using euclidean affine flower pollination algorithm (eFPA) and binary FPA (BFPA) in solar photo voltaic generation”, (2017) Renewable Energy, 107, pp. 550–566.

[16] Zhang W., Qu Z., Zhang K., Mao W., Ma Y., Fan X. “A combined model based on CEEMDAN and modified flower pollination algorithm for wind speed forecasting”, (2017) Energy Conversion and Management, 136, pp. 439–451.

[17] Barocio E., Regalado J., Cuevas E., Uribe F., Zúñiga P., Torres P.J.R. “Modified bio-inspired optimisation algorithm with a centroid decision making approach for solving a multi-objective optimal power flow problem”, (2017) IET Generation, Transmission and Distribution, 11 (4), pp. 1012–1022.

[18] Sarjiya, Putra, P.H., Saputra, T.A. “Modified flower pollination algorithm for nonsmooth and multiple fuel options economic dispatch”, (2017) Proceedings of 2016 8th International Conference on Information Technology and Electrical Engineering: Empowering Technology for Better Future, ICITEE 2016, art. no. 7863285.

[19] Salgotra R., Singh U. “Application of mutation operators to flower pollination algorithm”, (2017) Expert Systems with Applications, 79, pp. 112–129.

[20] Xu S., Wang Y., Huang F. “Optimization of multi-pass turning parameters through an improved flower pollination algorithm”, (2017) International Journal of Advanced Manufacturing Technology, 89 (1–4), pp. 503–514.

[21] Kyu-Ho Kim; Sang-Bong Rhee; Kyung-Bin Song; Lee, K.Y., An efficient operation of a microgrid using heuristic optimization techniques: Harmony search algorithm, PSO, and GA, 2012 IEEE Power and Energy Society General Meeting, pp.1,6, 22–26 July 2012.

[22] Patel M. R., 1999, “Wind and Solar Power Systems, Washington, D.C:CRC Press.

[23] I.I. Zahran (2012), investigating the Effect of Adding a PV system to the reliability of a large power system MSc. Thesis. Arab Academy for Science Technology and Maritime Transport.

[24] Chowdhury S., Chowdhury S.P. and Crossley P., Microgrids and Active Distribution Networks. London, United Kingdom: The Institution of Engineering and Technology, 2009.

[25] Caisheng Wang; Nehrir, H., Power management of a stand-alone wind/photovoltaic/fuel-cell energy system, 2008 IEEE Power and Energy Society General Meeting—Conversion and Delivery of Electrical Energy in the 21st Century, pp.1,1, 20–24 July 2008.

