# Supplementary material for: Novel optimization technique of isolated microgrid with hydrogen energy storage
Source: PLoS One. 2018 Feb 21;13(2):e0193224. doi: 10.1371/journal.pone.0193224 (PMC5821375; doi:10.1371/journal.pone.0193224)
Supplement: S1 File — (PDF) [file pone.0193224.s001.pdf]

Summer Day environmental data

Time	Wind Speed	Solar Irradiation	Temperature
1	3.1175	0	21
2	3.657739	0	21
3	3.657739	0	20
4	3.657739	0	20
5	3.966843	0	20
6	3.966843	0	19
7	4.585053	0	19
8	4.585053	0	19
9	5.306297	194.422	20
10	5.306297	469.616	22
11	6.491199	701.602	24
12	6.491199	874.571	25
13	6.903338	976.736	28
14	6.903338	1001.133	29
15	7.366995	946.101	31
16	7.366995	815.39	32
17	6.130577	617.907	33
18	6.130577	367.11	32
19	5.460849	80.092	31
20	5.460849	0	30
21	4.482018	0	27
22	4.482018	0	26
23	3.760774	0	25
24	3.760774	0	24

Winter Day environmental data

Time	Wind Speed	Solar Irradiation	Temperature
1	6.052692	0	16
2	5.59084	0	15
3	5.59084	0	15
4	5.688072	0	15
5	5.688072	0	15
6	6.32008	0	14
7	6.32008	0	13
8	7.225553	0	13
9	7.225553	263.41	14
10	7.584096	501.158	15
11	7.584096	686.033	17
12	7.225553	805.436	18
13	7.225553	851.23	18
14	6.593545	820.294	18
15	6.593545	714.736	18
16	4.788676	541.75	18
17	4.788676	313.125	18
18	4.606366	44.441	17
19	4.606366	0	17
20	5.688072	0	17
21	5.688072	0	16
22	5.59084	0	15
23	5.59084	0	15
24	6.052692	0	15
