# Supplementary material for: Novel optimization technique of isolated microgrid with hydrogen energy storage
Source: PLoS One. 2018 Feb 21;13(2):e0193224. doi: 10.1371/journal.pone.0193224 (PMC5821375; doi:10.1371/journal.pone.0193224)
Supplement: S2 File — (PDF) [file pone.0193224.s002.pdf]

Load Profiles Used in simulation:

Hour	Load (Summer)	Load (Winter)
1	2857.142857	810.8108108
2	2500	945.9459459
3	2357.142857	1081.081081
4	2214.285714	1486.486486
5	2214.285714	3513.513514
6	2500	4324.324324
7	2857.142857	3513.513514
8	3571.428571	3243.243243
9	4071.428571	2837.837838
10	4571.428571	2567.567568
11	4928.571429	2297.297297
12	4857.142857	2162.162162
13	4714.285714	2297.297297
14	4428.571429	2162.162162
15	4142.857143	1486.486486
16	3928.571429	1486.486486
17	4000	2297.297297
18	4285.714286	4594.594595
19	4428.571429	5000
20	4785.714286	4864.864865
21	4857.142857	4324.324324
22	4685.714286	3378.378378
23	4285.714286	3243.243243
24	3928.571429	3108.108108
